# The Effect of Wnt Pathway Modulators on Human iPSC-Derived Pancreatic Beta Cell Maturation

**DOI:** 10.3389/fendo.2019.00293

**Published:** 2019-05-08

**Authors:** Heidrun Vethe, Luiza Ghila, Magnus Berle, Laurence Hoareau, Øystein A. Haaland, Hanne Scholz, Joao A. Paulo, Simona Chera, Helge Ræder

**Affiliations:** ^1^Department of Clinical Science, KG Jebsen Center for Diabetes Research, University of Bergen, Bergen, Norway; ^2^Department of Surgery, Haukeland University Hospital, Bergen, Norway; ^3^Department of Clinical Medicine, University of Bergen, Bergen, Norway; ^4^Department of Global Public Health and Primary Care, University of Bergen, Bergen, Norway; ^5^Department of Transplant Medicine, Oslo University Hospital, Oslo, Norway; ^6^Institute of Basic Medical Sciences, University of Oslo, Oslo, Norway; ^7^Department of Cell Biology, Harvard Medical School, Boston, MA, United States; ^8^Department of Pediatrics, Haukeland University Hospital, Bergen, Norway

**Keywords:** human induced pluripotent stem cell, β-like cells, Wnt signaling pathway, tankyrase inhibition, *in vitro* maturation, proteomics, TMT11-plex, adult human islets

## Abstract

Current published protocols for targeted differentiation of human stem cells toward pancreatic β-cells fail to deliver sufficiently mature cells with functional properties comparable to human islet β-cells. We aimed to assess whether Wnt-modulation could promote the final protocol stages of β-cell maturation, building our hypothesis on our previous findings of Wnt activation in immature hiPSC-derived stage 7 (S7) cells compared to adult human islets and with recent data reporting a link between Wnt/PCP and *in vitro* β-cell maturation. In this study, we stimulated canonical and non-canonical Wnt signaling in hiPSC-derived S7 cells using syntetic proteins including WNT3A, WNT4, WNT5A and WNT5B, and we inhibited endogenous Wnt signaling with the Tankyrase inhibitor G007-LK (TKi). Whereas neither canonical nor non-canonical Wnt stimulation alone was able to mature hiPSC-derived S7 cells, WNT-inhibition with TKi increased the fraction of monohormonal cells and global proteomics of TKi-treated S7 cells showed a proteomic signature more similar to adult human islets, suggesting that inhibition of endogenous Wnt contributes toward final β-cell maturation.

## Introduction

Despite ongoing progress, it is at present still not possible to generate mature insulin-producing cells from human induced pluripotent stem cells (hiPSCs) that capture all aspects of endogenous β-cell *in vitro*. Cells generated with current published multi-step protocols (1–3) show elevated basal insulin secretion levels and insufficient glucose-responsiveness (4, 5), and are thus considered functionally immature (4). Additionally, *in vitro* differentiation leads to the generation of highly heterogeneous cell populations, largely composed of bi-hormonal (insulin+/glucagon+) cells alongside diverse categories of progenitor cells (6). It remains unclear to date, which signaling pathways will promote the last steps of β-cell differentiation and functional maturation, as well as whether these mechanisms can be specifically activated *in vitro*.

A candidate pathway contributing to β-cell maturation was recently suggested by Bader et al. (7), confirming a link between active non-canonical Wnt/planar cell polarity (PCP) signaling and *in vitro* β-cell maturation, as assessed in dispersed and re-aggregated post natal day 5 (P5) islet cells, pseudo-islets of Min6 insulinoma cells as well as in the human β-cell line (EndoC-β H1). The Wnt signaling pathways are a group of highly conserved pathways that regulate key aspects of cell fate decisions, migration, polarity, patterning and organogenesis during embryonic development (8–12). Previous studies have focused on the role of Wnt signaling in β-cell function (7, 13). Wnt signaling is highly conserved and serves as a stem cell niche signal in many contexts, as β-catenin is required to maintain an undifferentiated cell state (14, 15). In the pancreas, Wnt signaling is essential for pancreas development, islet function, and for the production and secretion of insulin in β-cells (16). Stage-specific signaling through Wnt regulates patterning and pancreas specification of human pluripotent stem cells, and canonical Wnt signaling has been found to induce a posterior endoderm fate and to enhance the development of pancreatic linage cells (17).

In our previous study comparing the proteome of S7 cells against the proteome of adult human pancreatic islets, we detected strong canonical and non-canonical Wnt pathway activation in S7 cells as compared to islets (18), suggesting that inhibition of the endogenous Wnt signaling could potentially promote the differentiation of S7 cells toward a more mature phenotype. Combined with recent data reporting that Wnt/PCP can trigger *in vitro* β-cell maturation (7), induced by WNT4 and WNT5A treatment, we hypothesized that Wnt modulation of S7 cells may affect their *in vitro* maturation potential. In this study, we expanded the selection of Wnt ligands to include WNT3A, WNT4, WNT5A, WNT5B, and WNT5A&5B combined. Moreover, to further test our hypothesis that inhibition of endogenous Wnt signaling drives S7 cells out of a progenitor state toward a more mature phenotype, we used the small molecule Tankyrase inhibitor G007-LK (TKi) to block endogenous Wnt signaling in S7 cell cultures.

## Materials and Methods

### Cell Source

In this study, we used human induced pluripotent cell (hiPSC) lines from healthy subjects obtained from three independent sources. The commercial control hiPSCs (ND41866) reprogrammed from fibroblasts using retroviral vectors (OCT4, SOX2, KLF4, CMYC) were purchased from Coriell Institute for Medical Research (Camden, NJ, USA). One line of normal healthy fibroblasts was reprogrammed by Sendai virus by Cellartis (Tekara Bio), and in parallel an additional fibroblast line from a normal healthy donor were episomal reprogrammed (in house) with plasmids (hUL, hSK, hOCT4/shp53), [27077 (pCXLE-hOCT3/4-shp53-F), 27078 (pCXLE-hSK) and 27080 (pCXLE-hUL)] all from Addgene. Human islets (*n* = 6 donors) were isolated as described previously (19) from pancreata obtained from three female and three male brain-dead deceased donors after informed consent from relatives for organ donation and for use in research at the islet isolation facility of Oslo University Hospital, Oslo, Norway. All hiPS cell lines were subjected to Stage-specific embryonic antigen 4 (SSEA4) enrichment (SSEA4 microbeads, MACS Miltenyi Biotec) before proceeding to differentiation. All iPSCs cell lines were confirmed to have normal karyotype, and to be mycoplasma free using a MycoAlert Mycoplasm Detection Kit (LT07-418, Lonza). All experiments with hiPSCs were approved by the Regional Committee of Medical and Health Research Ethics: hiPSCs (REK 2010/2295) and islets (REK 2011/426), and all methods were performed in accordance with the Helsinki Declaration.

### *In vitro* Differentiation Protocol

Three hiPSC lines were differentiated according to a seven-stage differentiation protocol (1) on Matrigel-coated plates for the duration of the whole differentiation protocol, without transfer to an air-liquid interface as we have reported previously (18). The cells were grown in S7 media for 7 days (S7d7 cells), and one well from each cell line was cultured for seven additional days in S7 media (S7d14 cells). hiPSC-derived cells were in parallel harvested for downstream immunofluorescence staining on coverslips from the same well as was harvested for quantitative proteomics analysis, at the end of S6d7, S7d7, and S7d14 days, respectively. Cells were also harvested 48 h after Wnt-treatment.

### Wnt Ligand Treatment of S7 Cells

S7 cells were in parallel stimulated with Wnt5A (645-WN, R&D Systems) (400 ng/mL), Wnt5B (7347-WN, R&D Systems) (400 ng/mL), a combination of Wnt5A/Wnt5B (400 ng/mL/80 ng/mL), Wnt4 (6076-WN, R&D Systems) (100 ng/mL), Wnt3A (5036-WN, R&D Systems) (200 ng/mL), and the tankyrase inhibitor G007-LK (5 μmol/L), for 4 h and kept 48 h in S7 differentiation media before harvest. Media was changed every day.

### Apoptosis Monitoring

For monitoring the apoptosis status of Wnt stimulated hiPSC-derived cells, and to find the time window for stimulation before induction of apoptosis, we used the Dead Cell Apoptosis kit with Annexin V and Propidium Iodide (PI) (V13241, Thermo Scientific). In brief, 1X annexin-binding buffer was prepared by adding 1 mL 5X annexin-binding buffer (Component C) to 4 mL MCDB131 basal medium. Working solution of PI (100 μg/mL) was prepared by diluting 5 μL of the 1 mg/mL PI stock solution (Component B) in 45 μL 1X annexin-binding buffer. Before changing media, each well was treated with 5 μL Alexa Fluor® 488 annexin V (Component A) and 1 μL 100 μg/mL PI working solution and cells were incubated at RT for 15 min. We removed all liquid and wash cells with 1X annexin-binding buffer (twice) and added the different WNT factors diluted in MCDB131 basal medium. Cells were monitored under a fluorescent microscope every 15 min for 4 h.

### Cell Viability

We used the NucleoCounter® NC-200™ for assessment of cell viability after Wnt-modulation using the vendors instructions.

### Static Incubation Glucose-Stimulated Insulin Secretion

Wnt treated and untreated S7 cells were rinsed twice with DPBS and then twice with Krebs buffer (129 mM NaCl, 5 mM NaHCO_3_, 4.8 mM KCl, 2.5 mM CaCl_2_, 1.2 mM MgSO_4_, 1 mM Na_2_HPO_4_, 1.2 mM KH_2_PO_4_, 10 mM HEPES, 0.1% BSA) in deionized water and then sterile filtered. The cells were pre-incubated in Krebs buffer spiked with 1.67 mM glucose (Krebs buffer low glucose) for 30 min. Media was collected after 30 min, centrifuged to remove unwanted cells and supernatant was transferred to a new vial. After low glucose incubation, the cells were incubated in Krebs buffer spiked with 20 mM glucose (Krebs buffer high glucose) for 30 min, and media was collected. Followed by incubation in Krebs buffer spiked with 20 mM glucose and 30 mM KCl for 10 min. All supernatants were frozen at −80°C until insulin levels were measured by ELISA (#10-1113-01; Mercodia).

### Immunofluorescence Staining

Cells were cultured on glass coverslips and fixed in 2% paraformaldehyde (PFA) for 15 min. The immunofluorescence protocol performed conformed to indications provided by the supplier. The following primary antibodies were used: mouse anti-β-catenin (1/500, ab22656, Abcam), rabbit anti-BMP4 (1/500, ab39973, Abcam), rabbit anti-ROR2 (1/50, ab92379, Abcam), rabbit anti-c-JUN (1/500, ab31419, Abcam), mouse anti-insulin (1/500, I2018, Sigma-Aldrich), guinea-pig anti-porcine insulin (1/500, A056401-2, Dako), mouse anti-porcine glucagon (1/500, G2654, Sigma-Aldrich), rat anti-somatostatin (1/100, sc-47706, Santa Cruz), guinea-pig anti-PDX1 (1/500, ab47308, Abcam), and rabbit anti-NKX6.1 (1/100, NBP1-82553, Novus), rabbit anti-MAFA (1/200, ab98859, Abcam). The following secondary antibodies were used: donkey anti-rabbit A488, donkey anti-rabbit A546, goat anti-mouse A568, chicken anti-rat A647, and goat anti-guinea-pig A647 (1/500, Molecular Probes). The nuclei were stained with DAPI (D1306, Molecular Probes). The samples were mounted in Prolong Diamond Antifade Mountant Media (P36970, Life technologies) and were analyzed with Leica TCS SP5 STED CW confocal microscope. No specific feature of the original data was obscured, eliminated or misrepresented.

### Cell Counting

From one coverslip, 20 snapshots were taken from different areas on the coverslip. From each snapshot all insulin+ and all glucagon+ cells were counted. Overlay of insulin+ and glucagon+ were used to count bi-hormonal cells.

### Global Proteomics Analysis

#### Cell Lysis and Protein Digestion

S6, S7d7, S7d14 and Wnt-modulated cell cultures were washed in DPBS and harvested with TrypLE™ Select Enzyme (1X) (12563011, Thermo Fisher Scientific), followed by centrifugation. Islets and hiPSC-derived cells were lysed as described previously (18). The protein concentration was determined using a BCA protein assay kit. Dry aliquots containing an estimated amount of 100 μg of proteins were further processed using Filter-Aided Sample Preparation (20). The six islet samples were combined and mixed to make a homogenous mixture, and 50 μg protein of the mix were aliqvoted into six separate samples for downstream TMT11-plex analysis.

#### Tandem Mass Tag (TMT) 11 Plex Labeling

TMT reagents were re-suspended in ACN. Desalted peptides were re-suspended in 50 μL of 200 mM EPPS pH 8.5, 15 μL of ACN, and 5 μL of the TMT reagents were added to the respective peptide samples, gently vortexed, and incubated for 1.5 h at RT. To prevent unwanted labeling, the reaction was quenched by adding 5 μL of 5% hydroxylamine and incubated for 15 min at RT. Equal amounts of the TMT-labeled samples were combined and concentrated to near dryness, followed by desalting via C18 solid phase extraction and passage over a Pierce detergent removal spin column (Thermo Fisher Scientific).

#### Off-Line Basic pH Reversed Phase Fractionation

The combined labeled peptide samples were pre-fractionated by basic pH reversed phase HPLC as described previously (21), using an Agilent (P/N 770995-902) 300Extend-C18, 5 μm, 250 × 4.6 mm id column, connected to an Agilent Technology off-line LC-system. Solvent A was 5% ACN, 10 mM NH_4_HCO_3_ pH8, and solvent B was 90% ACN, NH_4_HCO_3_ pH 8. The samples were re-suspended in 500 μL solvent A and loaded onto the column. Column flow was set to 0.8 mL/min and the gradient length was 70 min, as follows: from 0 to 35 min solvent 50% A/50% B, and from 35 to 50 min 100% B, and from 50 to 70 min 100% A. The labeled peptides were fractionated into 96 fractions, and further combined into a total of 12 fractions (22). Each fraction was acidified with 1% formic acid, concentrated by vacuum centrifugation to near dryness, and desalted by StageTip. Each fraction was dissolved in 5% ACN/ 5% formic acid for LC-MS/MS analysis.

#### LC-MS3 Analysis

From each of the 12 fractions, ~3 μg was dissolved in 1% aqueous formic acid (FA) prior to LC-MS/MS analysis on an Orbitrap Fusion mass spectrometer (Thermo Fisher Scientific, San Jose, CA) coupled to a Proxeon EASY-nLC 1000 liquid chromatography (LC) pump (Thermo Fisher Scientific). Peptides were fractionated on a 75-μm inner diameter microcapillary column packed with ~35 cm of Accucore resin (2.6 μm, 150 Å, Thermo Fisher Scientific, San Jose, CA). For each analysis, we loaded ~1 μg onto the column.

Peptides were separated using a 2.5 h gradient of 2–25% acetonitrile in 0.125% formic acid at a flow rate of ~350 nL/min. Each analysis used the multi-notch MS3-based TMT method (23) on an Orbitrap Fusion mass spectrometer, which has been shown to reduce ion interference compared to MS2 quantification (21). The scan sequence began with an MS1 spectrum (Orbitrap analysis; resolution 120,000; mass range 400–1,400 m/z; automatic gain control (AGC) target 5 × 10^5^; maximum injection time 100 ms). Precursors for MS2/MS3 analysis were selected using a Top10 method. MS2 analysis consisted of collision-induced dissociation (quadrupole ion trap analysis; AGC 2 × 10^4^; normalized collision energy (NCE) 35; maximum injection time 200 ms). Following acquisition of each MS2 spectrum, we collected an MS3 spectrum using our recently described method (23) in which multiple MS2 fragment ions were captured in the MS3 precursor population using isolation waveforms with multiple frequency notches. MS3 precursors were fragmented by high-energy collision-induced dissociation (HCD) and analyzed using the Orbitrap (NCE 65; AGC 2 × 10^5^; maximum injection time 300 ms, resolution was 50,000 fat 400 Th).

#### Data Analysis of MS-Data

Mass spectra were processed using a Sequest-based in-house software pipeline (24), and spectra were converted to mzXML using a modified version of ReAdW.exe. Database searching included all entries from the human uniprot database (March 11, 2014). This database was concatenated with one composed of all protein sequences in the reversed order. Searches were performed using a 50 ppm precursor ion tolerance for total protein level analysis. The product ion tolerance was set to 0.9 Da. These wide mass tolerance windows were chosen to maximize sensitivity in conjunction with Sequest searches and linear discriminant analysis (24, 25). TMT tags on lysine residues and peptide N termini (+229.163 Da) and carbamidomethylation of cysteine residues (+57.021 Da) were set as static modifications, while oxidation of methionine residues (+15.995 Da) was set as a variable modification.

Peptide-spectrum matches (PSMs) were adjusted to a 1% false discovery rate (FDR) (26, 27). PSM filtering was performed using a linear discriminant analysis, as described previously (24), while considering the following parameters: XCorr, ΔCn, missed cleavages, peptide length, charge state, and precursor mass accuracy. For TMT-based reporter ion quantitation, we extracted the summed signal-to-noise (S/N) ratio for each TMT channel and found the closest matching centroid to the expected mass of the TMT reporter ion.

The search space for each reporter ion was limited to a range of 0.003 Th to prevent overlap between the isobaric reporter ions. For protein-level comparisons, PSMs were identified, quantified, and collapsed to a 1% peptide false discovery rate (FDR) and then collapsed further to a final protein-level FDR of 1%. Moreover, protein assembly was guided by principles of parsimony to produce the smallest set of proteins necessary to account for all observed peptides.

Proteins were quantified by summing reporter ion counts across all matching PSMs using in-house software, as described previously (24). PSMs with poor quality, MS3 spectra with more than eight TMT reporter ion channels missing, MS3 spectra with TMT reporter summed signal-to-noise ratio that is less than 100, or no MS3 spectra were excluded from quantitation (28). Protein quantitation values were exported for further analysis in Microsoft Excel and GraphPad Prism (version 8). The mass spectrometry proteomics data have been deposited to the ProteomeXchange Consortium via the PRIDE (http://www.proteomexchange.org) partner repository with the dataset identifier PXD012081.

PC-plots were generated with default settings using the web based tool ClustVis (29). The pathway analyses were generated through the use of QIAGEN's Ingenuity Pathway Analysis (IPA®, QIAGEN Redwood City, www.qiagen.com/ingenuity). Briefly, the analyses were performed with the following settings: Expression Value Type (Exp Log Ration), Reference set (Ingenuity Knowledge Base + Endogenous chemicals), Relationships to consider (Direct and Indirect Relationships), Interaction networks (70 molecules/network; 25 networks/analysis), Data Source (all), Confidence (Experimentally Observed), Species (Human, Mouse, Rat), Tissue & Cell Lines (all), Mutations (all).

#### Statistics

R was used to normalize the data based on the common and homogenized adult human islet samples that were similar in each TMT11-plex run. Adult human islets (*n* = 2) in each TMT 11 plex experiment, in total *n* = 6 islet samples were analyzed. The normalized data was log2 transformed. As all samples were normalized to islets, the abundance of each protein from the islets will be equal to 1. R was also used for Euclidean distance measure on log2 scale between the different conditions and mean of islets. Two-sided unpaired *t*-test was performed GraphPad Prism (version 8), when specified. Results were expressed as mean with SEM and three data points were given corresponding to biological triplicates in each case the protein abundance were detected in all data sets. Statistical significance was considered *p* < 0.05.

## Results

**Figure 1:**
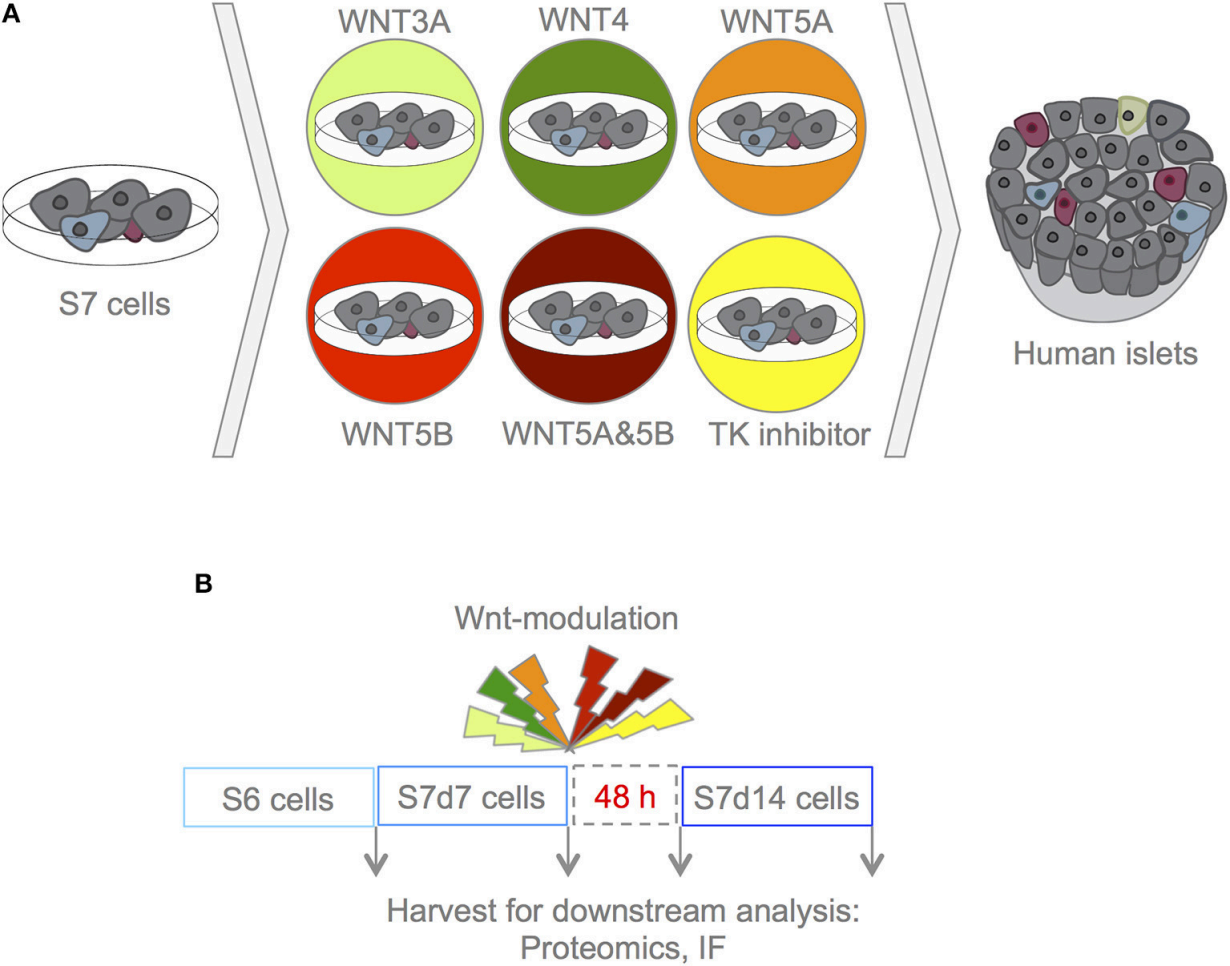
Experimental design. **(A)** With this experimental set-up we aimed to assess whether Wnt-modulation could drive maturation of S7 cells toward a phenotype that closer resembles that of β-cells as found in adult human islets. To assess the effects of Wnt-modulation of S7 cells, the Wnt-modulated cells were compared to un-stimulated S7 cells as well as to adult human islets. **(B)** S7d7 cell cultures were treated for 4 h with either WNT3A (light green), WNT4 (green), WNT5A (orange), WNT5B (red), a combination of WNT5A&5B (dark red) for stimulation of the canonical or non-canonical Wnt pathways, or TKi (yellow) to block endogenous Wnt signaling in S7d7 cell cultures. The Wnt-modulated S7 cells were maintained in differentiation culture for 48 h prior to harvest for downstream analysis [including proteomics analysis and immunofluorescence (IF)].

To investigate the role of Wnt modulation in hiPSC-derived S7 cells, three lines of hiPSCs, derived from three different human healthy subjects, generated through three different reprogramming methods, were differentiated using an established 7-stage differentiation protocol to produce S6 and S7 cells (corresponding to immature and maturing β-like cells, respectively) (1). S7 cell populations were maintained for 7 and 14 days, (here on S7d7 and S7d14 cells), respectively. S7 cells were treated with the selected Wnts and TKi for 4 h, and maintained in culture for an additional 48h before assessing the effect of Wnt-modulation on *in vitro* maturation. As negative and positive controls, respectively, we used un-stimulated S7 cells and adult human islets (Figure 1).

### Wnt-Modulation Did Not Compromise Cell Viability of hiPSC-Derived S7 Cells

For modulating Wnt-signaling in hiPSC-derived S7 cells, we chose to use similar concentrations as previously used for the WNT-ligands to stimulate Wnt-signaling in murine micro islets, human islets, as well as human β-cell line EndoC-BH1 (7). To ensure that Wnt-modulation did not induce apoptosis in our hiPSC-derived S7 cells, we assessed cell viability every 15 min by Annexin V and Propidium Iodide staining. Following the 4-h incubation, regardless of Wnt modulation, the S7 cells showed no membrane staining by Annexin V or nuclear staining for Propidium Iodide (data not shown).

To check for delayed reduction in viability, the viability of S7 cells was also checked at the moment of collection, i.e., 48 h after the Wnt-modulation (Supplementary Table 1). The overall viability of control untreated S7 cells was 89%. The WNT5B treated cells displayed an inversely correlated dose-dependent viability from 60% at 400 ng/mL to 71% at 80 ng/mL. In contrast, the viability of the S7 cells increased with the concentration of TKi from 53% at 1 μmol/L to 73%, respectively at 5 μmol/L. We chose to treat S7 cells with WNT3A (200 ng/mL), WNT4 (100 ng/mL), WNT5A (400 ng/mL), WNT5B (80 ng/mL), WNT5A&5B combination (400/80 ng/mL) and TKi at a concentration of 5 μmol/L. Overall, these results show that modulating the Wnt pathways in S7 cells for 4 h does not compromise cell viability, and causes only mild decrease in viability at later stages.

### Wnt Modulations of S7-Cells Show Heterogeneous Expression of Downstream Targets 48 h After Treatment

As we were aiming to assess whether Wnt-modulation could affect the *in vitro* maturation of hiPSC-derived insulin-producing cells, we did not harvest cells directly following the respective Wnt-treatments, but instead maintained the cells in S7 differentiation conditions for 48 h to promote maturation. First, we examined if the direct effect of Wnt-modulation persisted 48 h after treatment, by immunofluorescence (IF) and assessing the abundance of relevant proteins from our global proteomics analysis.

**Figure 2:**
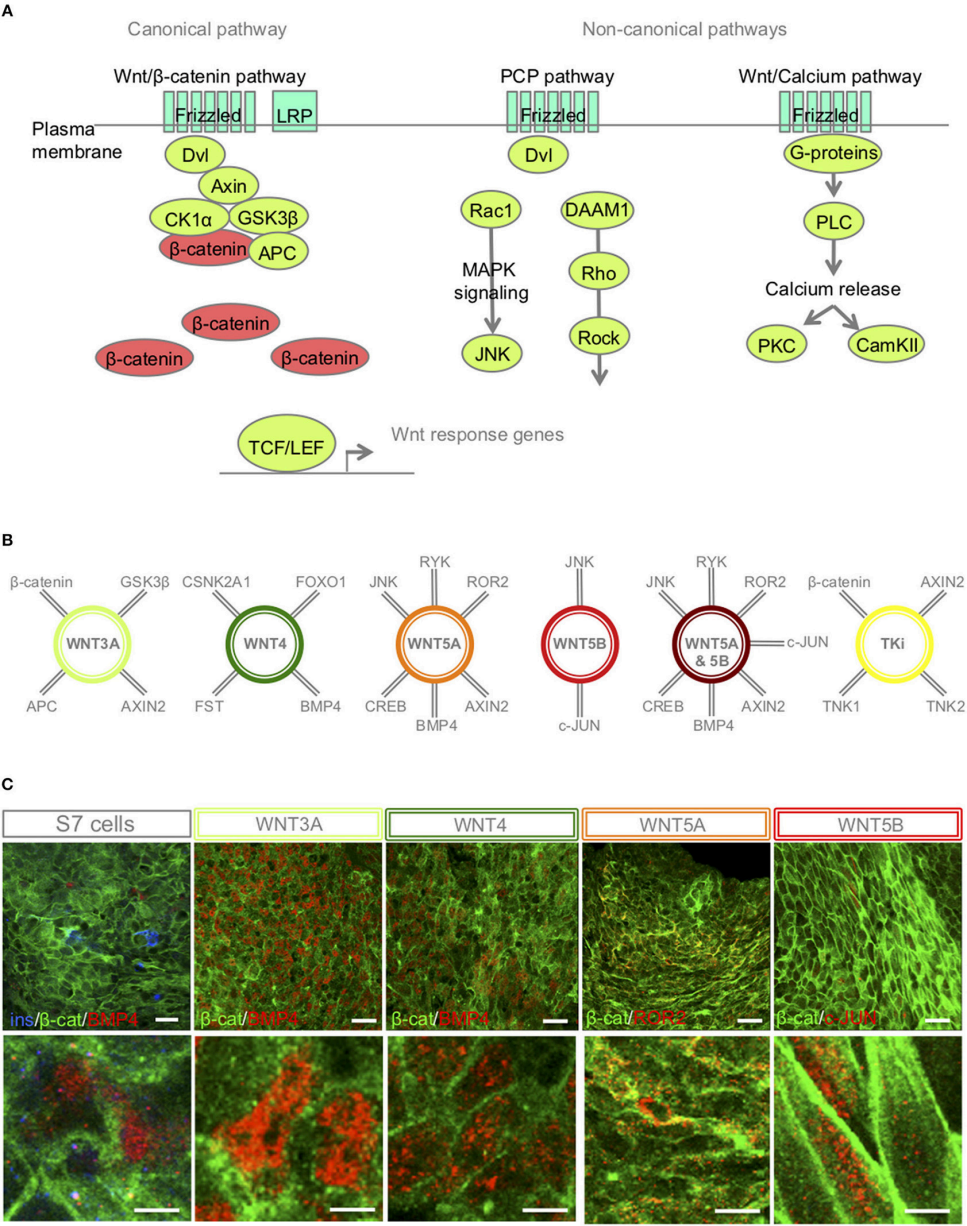
S7-cells show heterogeneous expression of downstream targets 48 h after Wnt-modulator treatment. **(A)** Schematic overview of key proteins of the canonical and non-canonical Wnt signaling pathways, in brief, Wnt ligand binds to its receptor Frizzled and co-receptors LRP5/6, receptor tyrosine kinase, (or ROR2) and transmits the signal via disheveled (Dvl) into the cytoplasm to activate the canonical Wnt pathway, or functions through non-canonical planar cell polarity (PCP) and Wnt/Ca^2+^. In the canonical Wnt signaling pathway, β-catenin accumulates in the cytoplasm and translocate to the nucleus to act as a transcription coactivator for the TCF/LEF transcription factor family. Without Wnt, β-catenin is degraded by the destruction complex, composed of Axin, adenomatosis polyposis coli (APC), glycogen synthase kinase 3β (GSK3β) and casein kinase 1α (CK1α). The non-canonical Wnt/PCP pathway is thought to use Ryk or ROR2 for activation; Dvl is recruited to form a complex with disheveled-associated activator of morphogenesis 1 (DAAM1). DAAM1 activates Rho that again activates Rho-associated kinase (ROCK). Dvl can also form a complex with Rac1 to activate JNK via the MAPK pathway. In the Wnt/Ca^2+^ pathway, binding of Wnt to Frizzled activates a trimetic G-protein leading to activation of PLC to cleave PIP2 to form DAG and IP3. IP3 binds to its receptor on the endoplasmic reticulum and calcium is released. Increased concentrations of calcium and DAG can again activate PKC and CaMKII. **(B)** A selection of interaction partners of the selected Wnt-ligands (30–33). **(C)** IF of β-catenin, BMP4, ROR2 and c-JUN in S7 cells, WNT3A, WNT4, WNT5A, WNT5B treated S7 cells, respectively. Scale bar 50 μm.

The canonical Wnt-ligand, Wnt3A, acts on target cells by binding to the frizzled receptors and LPR5/6 co-receptors, and induce accumulation of the un-phosphorylated form of β-catenin in the cytoplasm, making it available for entry into the nucleus, where the protein stimulates transcription of Wnt target genes (30) (brief overview of Wnt signaling pathways Figure 2A, interaction partners of the selected Wnt-ligands Figure 2B). In contrast, other Wnt ligands, such as Wnt4, Wnt5A and Wnt5B can activate non-canonical signaling cascades, such as Wnt/Ca^2+^ and PCP pathways (31) (Figure 2A).

IF revealed that most un-stimulated S7 cells were low BMP4 expressers with just small pockets of elevated expression. In contrast, both WNT3A and WNT4 stimulated cells show an increased BMP4 expression in the nucleus (Figure 2C). Dependent on receptor context [alternative Wnt-receptor ROR2, FZ4 or LRP5(32)], WNT5A can activate or inhibit Wnt signaling. It has been shown that in the presence of ROR2, WNT5A inhibits β-catenin signaling in a GSK3β-independent manner (33). Here our IF showed regions of ROR2 expression in the plasma membrane of WNT5A-stimulated S7 cells (Figure 2C). Furthermore, WNT5B can activate Wnt/β-catenin, Wnt/Ca^2+^ and PCP pathways, and regulates β-catenin and JNK. IF of WNT5B-treated S7 cells showed pockets of nuclear c-JUN expression (Figure 2C).

Independent of Wnt-modulation, IF revealed a heterogeneous expression of β-catenin in the membrane and the cytoplasm of the stimulated S7 cells (Figure 2C; in green) suggesting that the time point of β-catenin translocation was passed 48 h after Wnt-treatment, further suggesting that direct response to the relevant Wnt-ligands occurred prior to our time point of harvest of S7 cells. Wnt-downstream target proteins were also heterogeneously expressed among the S7 cell population (Figure 2C). An explanation for this mosaic pattern is the already known high heterogeneity of the S7 cell population itself (6). Due to this intra-sample variation, our bulk proteomic assay could potentially not detect significant differences in these specific protein abundances between different S7 samples.

Of note, previous findings show that WNT4 induced JNK activation in the EndoC-β H1 cell line (7). These findings, however, fail to compare this increased expression with its natural expression in human islets or isolated β-cells. Interestingly, our proteomics data revealed that untreated S7 cells already exhibit higher JNK levels as compared to adult human islets, suggesting that any further up-regulation by WNT4 treatment will push these cells even further away from their β-like molecular fingerprint goal (Supplementary Figure 1A). A similar example is JUN, displaying similar levels between un-stimulated S7 cells and islets, which upon Wnt-treatment were reduced (Supplementary Figure 1A).

Taken together these results show that very few direct markers of Wnt-pathway modulations persisted 48h after the treatment. However, our aim of this study was to assess the influence of Wnt-modulation on *in vitro* maturation of S7 cells.

### Wnt-Modulation Influences the Heterogeneity of the S7 Cell Population

**Figure 3:**
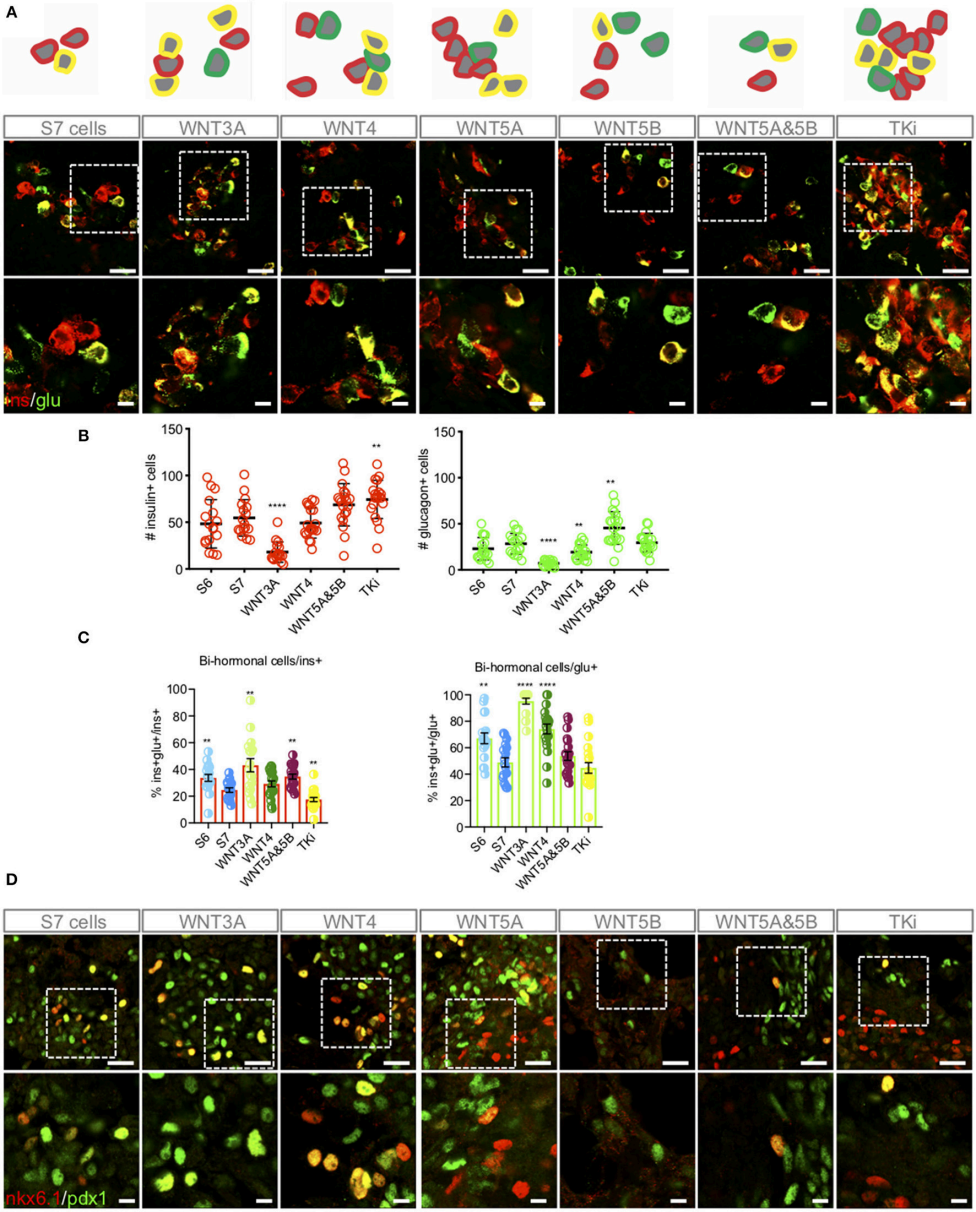
Wnt-modulation influences the distribution of mono-hormonal and bi-hormonal S7 cells. **(A)** Representative schematic drawing and IF analysis for insulin+ (red), glucagon+ (green) cells and insulin+/glucagon+ cells (yellow) of the respective S7 cell populations. **(B)** Calculations of insulin+ (red, left) and glucagon+ (green, right) cells were done as described in Methods. The y-axis shows the number of insulin+ and glucagon+ cells, respectively. The figure shows standard error of the mean (SEM) values for each of the columns in bar charts. ^**^P < 0.006, ^****^P < 0.0001 vs. S7 cells with two-tailed *t*-test. No significant comparisons show no stars. The number of insulin+ cells and glucagon+ cells were normalized to total cell count (dapi+ cells) see Supplementary Figure 2. **(C)** Overlay of insulin+ and glucagon+ were used to count bi-hormonal cells, in which bi-hormonal (ins+glu+) cells were calculated as a fraction of insulin+ (red bar chart) and glucagon+ (green bar chart) cells, respectively. The figure shows standard error of the mean (SEM) values for each of the columns in bar charts. ^**^P < 0.006, ^****^P < 0.0001 vs. S7 cells with two-tailed, type two *t*-test. No significant comparisons show no stars. **(D)** IF analysis of NKX6.1+ (red), PDX1+ (green) cells and NKX6.1+/PDX1+ cells (yellow). Scale bar upper panel: 25 μm, lower panel: 7.5 μm.

The islets of Langerhans contain five different endocrine cell types such as glucagon-secreting α-cells, insulin-secreting β-cells, somatostatin-secreting δ-cells, pancreatic polypeptide-secreting PP-cells and ghrelin-secreting ε-cells. Differentiation of hiPSCs to achieve mono-hormonal β-like cells that display controlled glucose-responsiveness has been proven to be difficult to achieve (4). As reported previously (6), we have also experienced that S7 cells display a high and unavoidable degree of heterogeneity with few mono-hormonal insulin+ cells, and many bi-hormonal insulin+/ glucagon+ cells, as well as mixtures of the transcription factors Pancreatic and duodenal homeobox 1 (PDX1) and homeobox protein NKX6.1, i.e., NKX6.1+/PDX1+, NKX6.1+/PDX1– and NKX6.1–/PDX1+ cells (Figures 3A, D). By comparing S6 cells corresponding to immature β-like cells (1) to S7 cells, we found as expected that the level of bi-hormonal cells decreased during *in vitro* maturation of hiPSC-derived cells (Figure 3C). Following Wnt-modulations, TKi induced the expression of islet-like groups of insulin+ cells, and displayed the significantly highest fraction of insulin+ cells and significantly lowest fraction of bi-hormonal cells to mono-hormonal cells as compared to the other Wnt-modulators (Figures 3A–C) and to un-stimulated S7 cells. In contrast, WNT3A treated S7 cells displayed the highest fraction of bi-hormonal cells and the lowest numbers of insulin-only expressing cells (Figures 3B,C, Supplementary Figure 2). Interestingly, most of the glucagon+ cells (95%) in the WNT3A-treated S7 cells were bi-hormonal, in which few single-hormonal glucagon+ cells were found (Figure 3C). WNT4 treated S7 cells included many individually located mono-hormonal insulin+ cells, but a higher fraction of bi-hormonal cells compared to TKi-stimulated cells. The combined stimulation of WNT5A&5B increased the number of both mono-hormonal insulin+, mono-hormonal glucagon+ cells (Figure 3B), as well as the fraction of bi-hormonal cells as compared to un-stimulated cells. We also checked IF of PDX1 and NKX6.1, but did not detect WNT-dependent alterations in PDX1/NKX6.1 expression, although we noted that Wnt-modulation led to extensive heterogeneity with mixtures of NKX6.1+/PDX1+, NKX6.1+/PDX1– and NKX6.1–/PDX1+ cells (Figure 3D).

### Wnt-Modulation of S7 Cells Leads to Altered Levels of Pancreatic Endocrine Proteins

**Figure 4:**
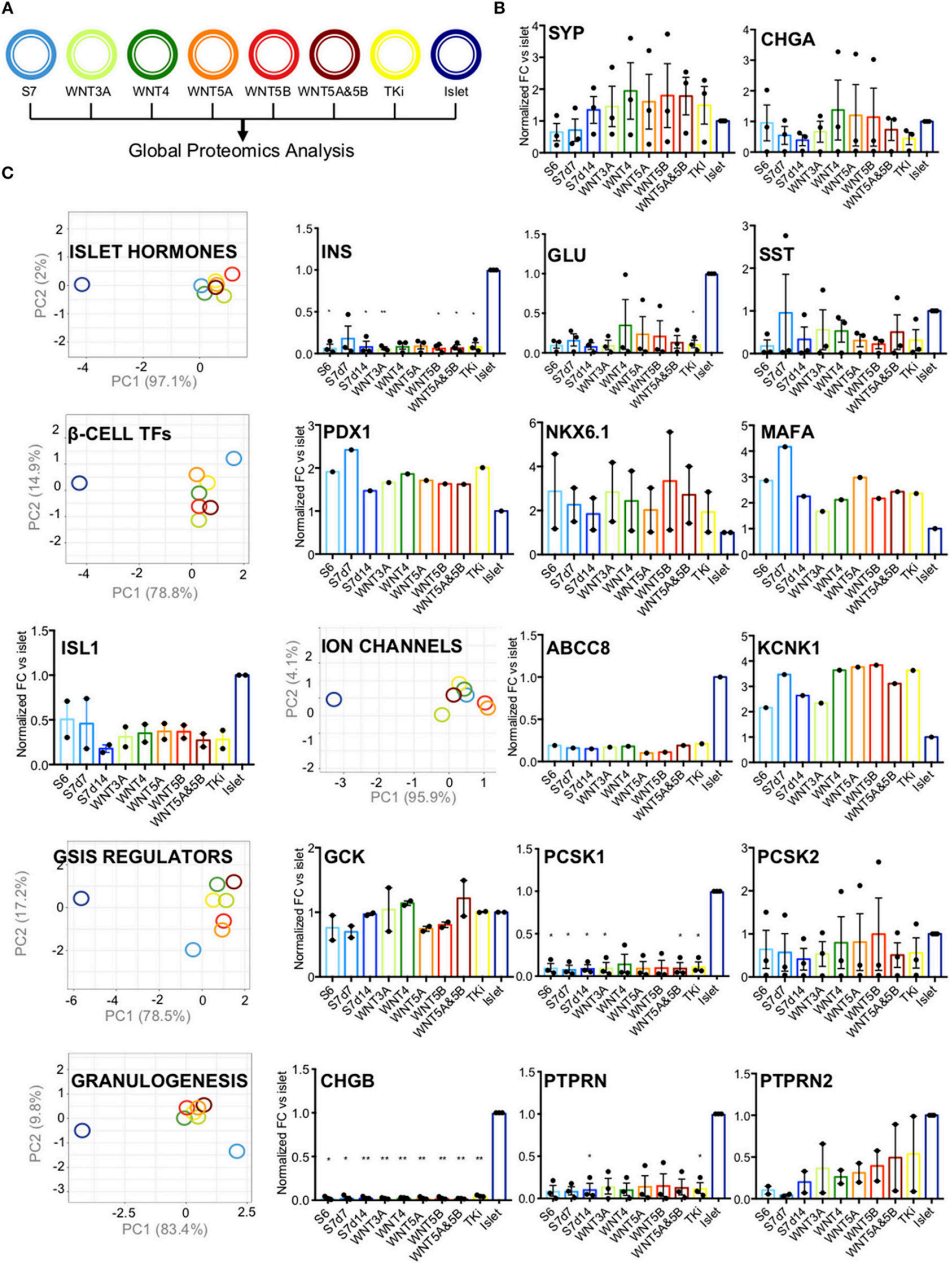
Pancreatic endocrine markers. **(A)** Schematic drawing of the color code for the different samples analyzed. **(B)** Protein abundance of pan-endocrine markers Synaptophysin (SYP) and Chromogranin (CHGA) in S6, S7d7, S7d14, Wnt-modualted S7 cells and adult human islets. The y-axis shows normalized fold change vs. islets for each protein. The data shown is normalized protein levels from all cell lines (*n* = 3, when detected) and is shown as mean with SEM. **(C)** PC-plots for hormones (INS, GCG, SST), transcription factors (PDX1, NKX6.1, MAFA, and ISL1), ion channels (ABCC8, KCNK1), GSIS regulators (GCK, PCSK1, PCSK2, LDHA, HK1, HK2, ALDOB) and proteins involved in granulogenesis (CHGA, CHGB, PTPRN, PTPRN2). Unit variance scaling is applied to rows. Singular value decomposition(SVD) with imputation is used to calculate principal components. X and y-axis show principal component 1 and principal component 2 that explain the % of the total variance, respectively. *N* = 8 data points. Also included are charts showing normalized fold change (y-axis) for each protein vs. islets. The data shown is normalized protein levels from all cell lines (*n* = 3, when detected) and is shown as mean with SEM. ^*^P < 0.05, ^**^P < 0.006 as vs. islets (normalized abundance level of adult human islets as reference) *t*-test. No significant comparisons show no stars.

We next investigated the effects of Wnt-modulation of S7 cells on protein level based on quantitative proteomics assessment (Figure 4A). First, we assessed the abundance of pan-endocrine markers Synaptophysin (SYP) and Chromogranin A (CHGA) (Figure 4B). For SYP we observed increased levels after Wnt-modulation, both after Wnt-stimulation and -inhibition (TKi). Whereas some Wnt-treatments yielded SYP levels close to the islet levels, other Wnt-treatments led to even higher levels than islet levels. CHGA levels were not significantly altered, suggesting that modulating the Wnt pathway has no effect on its regulation.

Further, we grouped the selected markers according to their role in β-cell function, with groups of islet hormones, of typical mature β-cell transcription factors, ion channels, proteins involved in the regulation of glucose-stimulated insulin secretion (GSIS), and of proteins involved in granulogenesis (PC-plots and relative abundance charts Figure 4C, respectively). As expected, the levels of pancreatic hormones (INS, GLU and SST) were lower than in adult human islets, and none of the selected Wnt-modulation led to islet hormone levels closer to that of human islets (Figure 4C).

The transcription factors PDX1, NKX6.1 and pancreatic β-cell-specific transcriptional activator MAFA are markers of adult human β-cells (34), together with insulin gene enhancer protein ISL1. Wnt-modulation had a moderate effect on the expression β-cell transcription factors (PDX1, NKX6.1, MAFA, and ISL1), which exhibited intermediate PC1 values between islets and un-stimulated S7 control cells (Figure 4C). WNT4 has previously been reported to increase the levels of NKX6.1 and PDX1 protein in the human EndoC-β H1 β-cell line, and human islets (7). Based on our proteomics data, the protein levels of NKX6.1, PDX1, and MAFA showed an opposite pattern with higher levels in both S6 and S7 cells as compared to adult human islets (protein plots in Figure 4C). Wnt-modulation of S7 cells seemed to reduce the protein level of PDX1 and MAFA, while NKX6.1 was increased by WNT3A, WNT4, WNT5B, and the WNT5A&5B combination. Whereas TKi did not change the NKX6.1 protein levels as compared to S7 cells. The protein abundance of ISL1 was minimally affected by Wnt-treatment.

To further characterize our S7 cells response to the different Wnt-modulations we assessed the various components of the glucose sensing machinery. We found no clear effects by Wnt-perturbations on the protein levels of ion channels (ABCC8 (SUR1) and KCNK1) (Figure 4C) or the levels of glycolytic enzymes (GCK, PCSK1, PCSK2, HK1, HK2, LDHA, ALDOB) (PC-plot and protein plots in Figure 4C; protein plots for HK1, HK2, LDHA, ALDOB in Supplementary Figure 1A) or proteins involved in granulogenesis (CHGA (Figure 4B), CHGB, PTPRN, PTPRN2). Moreover, to test the effect of Wnt-modulation on the functionality of the S7 cells we performed a static glucose-stimulated insulin secretion assay. This showed that neither of the WNT modulators improved or altered glucose stimulated insulin secretion of S7 cells (Supplementary Figure 1B), and fold difference in insulin secretion (Supplementary Figure 1C). In short, these results suggest that Wnt-treatment did not change the levels of essential proteins involved in the fine-tuned regulation of insulin secretion, although the grouped transcription protein levels displayed a tendency toward the protein levels of human islets.

### Inhibition of Endogenous Wnt Signaling by TKi Brings S7 Cellular Proteome Closer to That of Adult Human Islets

**Figure 5:**
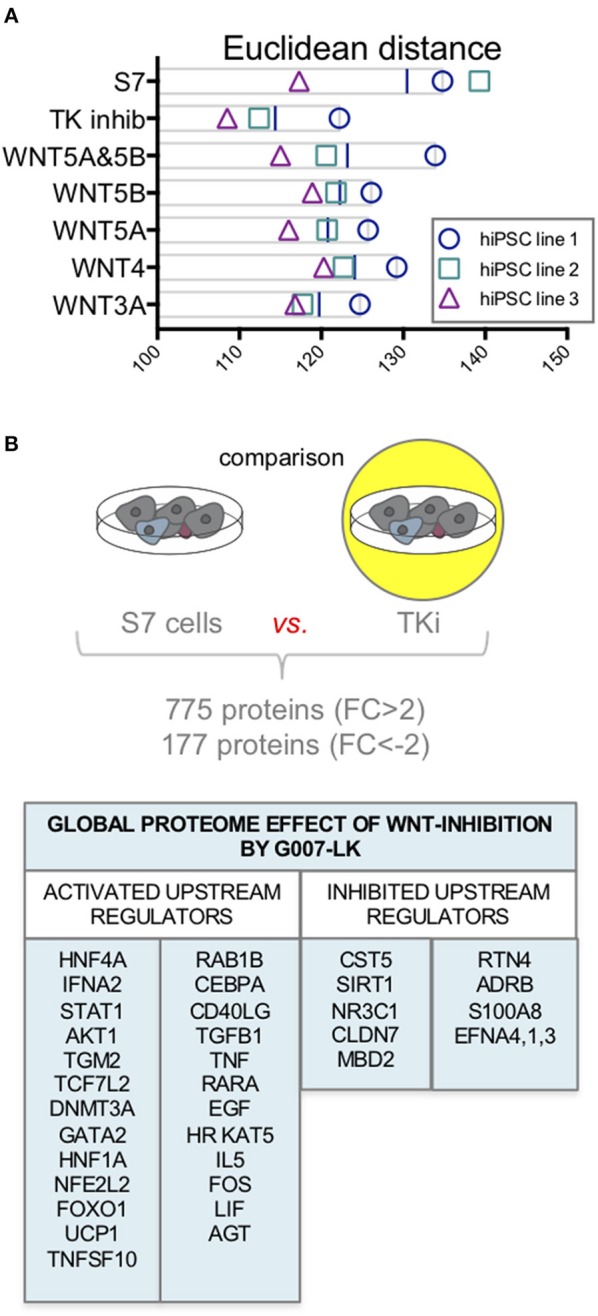
Proteome changes in response to Wnt-modulation of S7 cells. **(A)** Euclidean distance on log2 scale between S7 cells and the different Wnt-modulated S7 cell conditions and adult human islets. Of the three cell lines (*n* = 3, biological triplicates), TKi had the lowest distance measured between TKi-treated S7 cells vs. adult human islets. **(B)** Using quantitative proteomics data from one of the cell lines we examined the global proteome effect of endogenous Wnt inhibition by TKi on S7 cells resulting in increased abundance of 755 proteins (FC > 2) and decreased abundance of 177 proteins (FC < −2). The IPA software predicted upstream regulators protein signature based on these differentially regulated proteins.

Although assessment of specific β-cell proteins did not alter with Wnt-modulation toward the levels in human islets, we aimed to assess if there were any Wnt-modulating effects driving the global proteome of S7 cells toward an adult human islet-fate. To assess this, we compared all protein abundances of the adult human islet proteome (*n* = 9,602 proteins) to that of S7 cells stimulated with the selected Wnt-ligands (WNT3A, WNT4, WNT5A, WNT5B, and WNT5A&5B) or TKi. We calculated the Euclidean distance on log2 scale between S7 cells, each Wnt-modulated S7 cells and adult human islets. Inhibition of endogenous Wnt signaling by TKi had the lowest distance measured as compared to adult human islets for the three cell lines (Figure 5A).

To evaluate the effect of inhibiting endogenous Wnt signaling by TKi in S7 cells, we first assessed the activation status of the Wnt/β-catenin, Wnt/Ca^2+^ and Wnt/PCP pathways using the IPA software. IPA predicted negative activation z-scores for Wnt/β-catenin, Wnt/Ca^2+^ and Wnt/PCP pathways in TKi-treated S7 cells compared to untreated S7 cells, suggesting inhibited pathway activity in S7 cells as an effect of Tankyrase inhibition (Supplementary Table 2).

To further assess the global proteome effect of TKi inhibition of S7 cells, we compared the proteome of TKi-treated S7 cells to the proteome of S7 cells from one of the cell lines, resulted in increased abundance of 772 proteins (FC > 2) and decreased abundance of 174 proteins (FC < −2). To investigate the biological significance of these proteins, we used the IPA software. Based on the protein abundance profiles [946 proteins (2 < FC < −2)], IPA predicted a signature of upstream regulators for TK inhibition of S7 cells (Figure 5B). Among the predicted activated upstream regulators, we found HNF4A, STAT1, AKT1, DNMT3A, HNF1A, and FOXO1. CST5 and SIRT1, a regulator of TP53 and FOXO1 were among the upstream regulators predicted as inhibited.

## Discussion

In this study, we are the first to report a comprehensive characterization study of the effects of Wnt-modulation on *in vitro* maturation of hiPSC-derived S7 cells toward the profile of adult human islets. We found that Wnt-inhibition significantly increased the fraction of mono-hormonal S7 cells and reduced the fraction of bi-hormonal cells, whereas Wnt-stimulation did not affect these ratios. Wnt-inhibition also increased the similarity of the global proteomic signature of S7 cells and human islets, whereas singular proteomics-based maturation markers and glucose-stimulated insulin secretion were unchanged.

Previous studies investigating Wnt signaling in β-cells (7, 13, 16) have used variable time windows for the perturbation of Wnt signaling, ranging from 4 h to 4 days. Also the maintenance in culture following Wnt-modulation has varied. Nostro et al. stimulated pancreatic endoderm cell populations with WNT3A and the Wnt inhibitor for 3 days (17), following culture in four additional days to promote maturation, in which WNT3A increased INS expression. Bader et al. stimulated micro islets and human islets for 4 days with WNT4, and the human β-cell line EndoC-BH1 for 4 h and 4 days (7), in which WNT4 increased the expression of PDX1 and NKX6.1 in the EndoC-BH1 cells as well as human islets. However, none of these studies reported apoptosis monitoring which could potentially bias the results. In our studies of Wnt-perturbation of S7 cells, we observed no induction of apoptosis occurring before 4 h, nor at the moment of collection.

In the pancreas, Wnt signaling has several important roles in pancreatic development and islet functions (16), and previous studies have focused on the role of Wnt signaling in β-cells (7, 13, 16). Stage-specific signaling through Wnt regulates patterning and pancreas specification of hPSCs, and canonical Wnt signaling has been found to promote the development of pancreatic linage cells (17). We recently reported strong Wnt activation to be characteristic of immature hiPSC-derived S7 cells when compared to adult human islets (18). Our previous findings are in agreement with Qiu et al., in which single-cell RNA-seq analyses of mouse β-cell from fetal to adult stage of development, identified enrichment of Wnt signaling pathways in immature compared to mature β-cells (35). In contrast to P5 murine islet cells, Min6 cells and the adult human β-cell line EndoC-BH1(7), we found that modulation of non-canonical Wnt signaling did not improve GSIS of S7 cells. Contradicting previous findings of WNT4 increasing levels of NKX6.1 and PDX1 protein in the human EndoC-β H1 β-cell line (7), we found that WNT4 reduced the protein levels of PDX1and MAFA as compared to un-stimulated S7 cells. However, WNT4 affected the protein levels of these β-cell transcription factors to become more similar to the protein levels of adult human islets. Surprisingly, NKX6.1 and MAFA levels were lower in human islets (positive control) compared to un-stimulated S7 cells (negative control). We believe this is caused by the islet being a mixture of several different cell types diluting the signals of the low-abundant transcription factors. A previous report indicated that WNT3A stimulation (and not DKK1 stimulation) induced INS expression but not PDX1 expression (17). We, however, report unaltered INS and PDX1-expression.

To test our hypothesis that inhibition of endogenous Wnt signaling promotes β-cell-like maturation of S7 cells, we used the small molecule Tankyrase inhibitor to block endogenous Wnt signaling in S7 cell cultures. TKi acts inhibitory by promoting formation of membrane-free structures containing active components of the WNT/β-catenin destruction complex in colorectal cancer cell lines (36). TKi has been reported to decrease Wnt signaling in colon carcinoma cells (37), but the effect of tankyrase inhibition of hiPSC-derived S7 cells has to our knowledge never been investigated. We found that TKi-treated S7 cells increased the fraction of insulin+ cells significantly, and at the same time reduced the fraction of bi-hormonal cells significantly as compared to untreated S7 cells. Moreover, the global proteome signature of TKi-treated S7 cells was more similar to that of adult human islets, as compared to stimulatory Wnt-modulations and un-treated S7 cells. Finally, activation scores for Wnt/β-catenin, Wnt/Ca^2+^ and Wnt/PCP pathways indicated that all these pathways were inhibited by TKi. Taken together, these findings suggest that inhibition of both endogenous canonical and non-canonical Wnt-signaling pathways via tankyrase inhibition may promote maturation of S7 cells. However, using various TK inhibitors (i.e., G-631, XAV939, JW55) (36) or other Wnt inhibitors (i.e., DKK) (17) with differences in their specificities (i.e., affecting either canonical or non-canonical signaling) would probably even better define to which degree each of the canonical or non-canonical signaling pathways contributes to beta cell maturation, and whether selective inhibition would exert an even larger effect. In addition, we assessed a heterogenous pool of unsorted S7 cells potentially diluting an underlying larger effect on maturation. Sorting beta cells (38, 39) would probably enrich the population of studied S7 cells to yield potentially maturation effects of a greater magnitude, but our lines lacked sortable reporters analogous to the Mel1 Ins-GFP reporter lines used by others (40), and there are to our knowledge no sorting procedures established for stem-cell derived S7 cells. Further analysis of upstream regulators predicting the molecular proteomic signature overlapping proteins of TKi-modulated S7 cells and human islets indicated several proteins with relevance for β-cell maturation, most notably DNA methyltransferase (DNMT3A). DNMT3A plays a key role in β-cells maturation after birth, in which the protein represses *Ldha, Hk1*, and *AldoB*, and thereby enable the coupling of insulin secretion to glucose levels, which results in GSIS (41). β-cell repression of these key metabolic genes is essential toward maintenance of a functional mature phenotype. Our results suggest that TKi modulation of S7 cells changed the proteome comparable to what would have been observed by activation of DNMT3A.

The inherent relative low efficiency of targeted differentiation and striking heterogeneity of cells using current protocols combined with extensive cell line variability clearly limits reproducible analysis of maturity and functionality of β-like cells derived from hiPSC and hESCs. Although there are clear advances in protocols for generating hPSC-derived β-like cells with a partly functional and mature phenotype (1–3), these published sequences have been optimized for specific hESC lines and lacks optimization for other stem cell lines, in particular for hiPSCs.

As we have reported previously (18), direct comparison of the proteome of S7 cells to adult human islets, is required to characterize β-cell maturity as an addition to evaluating existing markers and functional assays. As reported previously by Nostro et al, endogenous BMP signaling varies substantially between different hESC lines and can dramatically affect the outcome of the pancreatic differentiation protocol (17). In this current study, we addressed variability by reporting the results from analysis of three lines of hiPSCs, derived from three different human healthy subjects, generated though three different reprogramming procedures. The efficiency of the differentiation varied between hiPSC lines, but also the response to the different Wnt-modulations. As previously described (6), S7 cells displayed a high and unavoidable degree of heterogeneity with few mono-hormonal insulin+ cells, and many bi-hormonal insulin+/ glucagon+ cells. The S7 cellular response to Wnt-modulations also displayed heterogeneous expression of BMP4, β-catenin, ROR2, c-JUN, PDX1, NKX6.1, as well as INS and GCG. We believe that optimizing current targeted differentiation protocols for a larger range of different stem cell lines, also including hiPSCs, will substantially improve reproducibility and allow for more robust studies of the role of specific pathways in β-cell maturation.

In conclusion, we have comprehensively assessed the role of Wnt-modulation in the final maturation stage of pancreatic β-like cells using a current state-of-the art targeted differentiation protocol. Our results indicate that Wnt-inhibition may modestly improve β-cell maturation.

## Ethics Statement

All experiments with hiPSCs were approved by the Regional Committees for Medical and Health Research Ethics: hiPSCs (REK 2010/2295) and islets (REK 2011/426), and all methods were performed in accordance with the Helsinki Declaration.

## Author Contributions

HV, LG, SC, and HR contributed with conception and design of the experiments. HV, MB, and LH performed the experiments. HV, ØH, and SC analyzed the data. JP mass spectrometry analysis. HS provided adult human islets. HV, SC, and HR wrote the first draft. All authors approved the final version of the manuscript.

### Conflict of Interest Statement

The authors declare that the research was conducted in the absence of any commercial or financial relationships that could be construed as a potential conflict of interest.

## Supplementary Material

Supplementary Figure 1 **(A)** Protein levels of JNK and JUN in the Wnt-modulated S7 cells detected by quantitative proteomics. The y-axis shows normalized fold change vs. adult human islets. Data presented normalized protein levels from all three cell line (*n* = 3) and is shown as mean with SEM. ^*^*P* < 0.05, vs. islets, with two-tailed, type two *t*-test. **(B)** Protein charts for proteins involved in GSIS regulation (LDHA, HK1, HK2, ALDOB). **(C)** Insulin secretion of S7d7 cells and Wnt-modulated S7 cells after static incubation in 1.67 mM glucose (low glucose) for 30 min, 20 mM glucose (high glucose) for 30 min, and 20 mM glucose supplemented with 30 mM KCl (high glucose + KCl) for 10 min. The insulin levels were normalized to the total cell number in each well for each condition. *N* = three hiPSC-derived cell lines. The data is shown as mean with SEM. ^*^ marks significant GSIS as compared to S7 cells, no significant results were obtained.

Supplementary Figure 2 Dapi+ cells were counted from three confocal areas for each condition to calculate the % insulin+ and % glucagon + cells as normalized to total number of dapi+ cells.

Supplementary Table 1 The viability of S7 cells was also checked 48 h after Wnt-treatment. We chose to treat S7 cells with WNT3A (200 ng/mL), WNT4 (100 ng/mL), WNT5A (400 ng/mL), WNT5B (80 ng/mL), WNT5A&5B combination (400/ 80 ng/mL) and TKi at a concentration of 5 μmol/L). The table shows the viability of S7 cells at each of the concentrations of Wnt-modulators tested.

Supplementary Table 2 Activation status of Wnt/β-catenin, Wnt/Ca^2+^, and Wnt/PCP pathways in TKi-treated S7 cells as compared to untreated S7 cells, The significance values for the canonical pathways is calculated using Fisher's exact test and assuming a right-tailed distribution (-log(*p*-value)). The significance indicates the probability of association of proteins from our dataset with proteins of the canonical pathway by chance alone. Ratio denotes here the number of proteins from our data set annotated as involved in the pathway as a fraction of all proteins annotated in the given pathway. IPA predict activated pathways (positive z-scores) or inhibited pathways (negative z-scores) based on the relative abundance of all proteins annotated in Wnt pathways from our dataset.
