# Supplementary figures and images for: The Effect of Wnt Pathway Modulators on Human iPSC-Derived Pancreatic Beta Cell Maturation
Source: Front Endocrinol (Lausanne). 2019 May 8;10:293. doi: 10.3389/fendo.2019.00293 (PMC6518024; doi:10.3389/fendo.2019.00293)

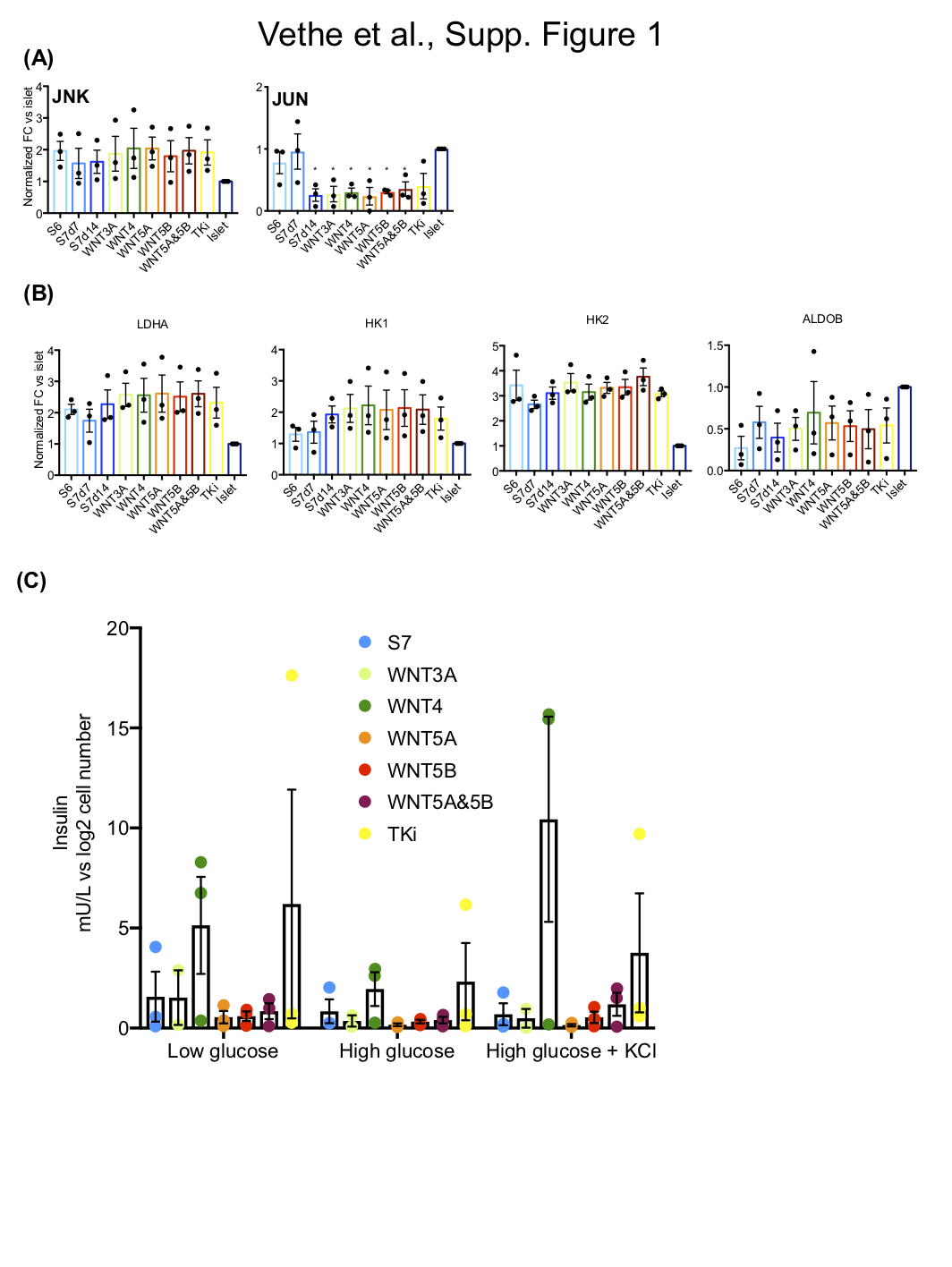

Supplement: Supplementary Figure 1 — (A) Protein levels of JNK and JUN in the Wnt-modulated S7 cells detected by quantitative proteomics. The y-axis shows normalized fold change vs. adult human islets. Data presented normalized protein levels from all three cell line (n = 3) and is shown as mean with SEM. *P < 0.05, vs. islets, with two-tailed, type two t-test. (B) Protein charts for proteins involved in GSIS regulation (LDHA, HK1, HK2, ALDOB). (C) Insulin secretion of S7d7 cells and Wnt-modulated S7 cells after static incubation in 1.67 mM glucose (low glucose) for 30 min, 20 mM glucose (high glucose) for 30 min, and 20 mM glucose supplemented with 30 mM KCl (high glucose + KCl) for 10 min. The insulin levels were normalized to the total cell number in each well for each condition. N = three hiPSC-derived cell lines. The data is shown as mean with SEM. * marks significant GSIS as compared to S7 cells, no significant results were obtained. [file Image_1.TIFF]

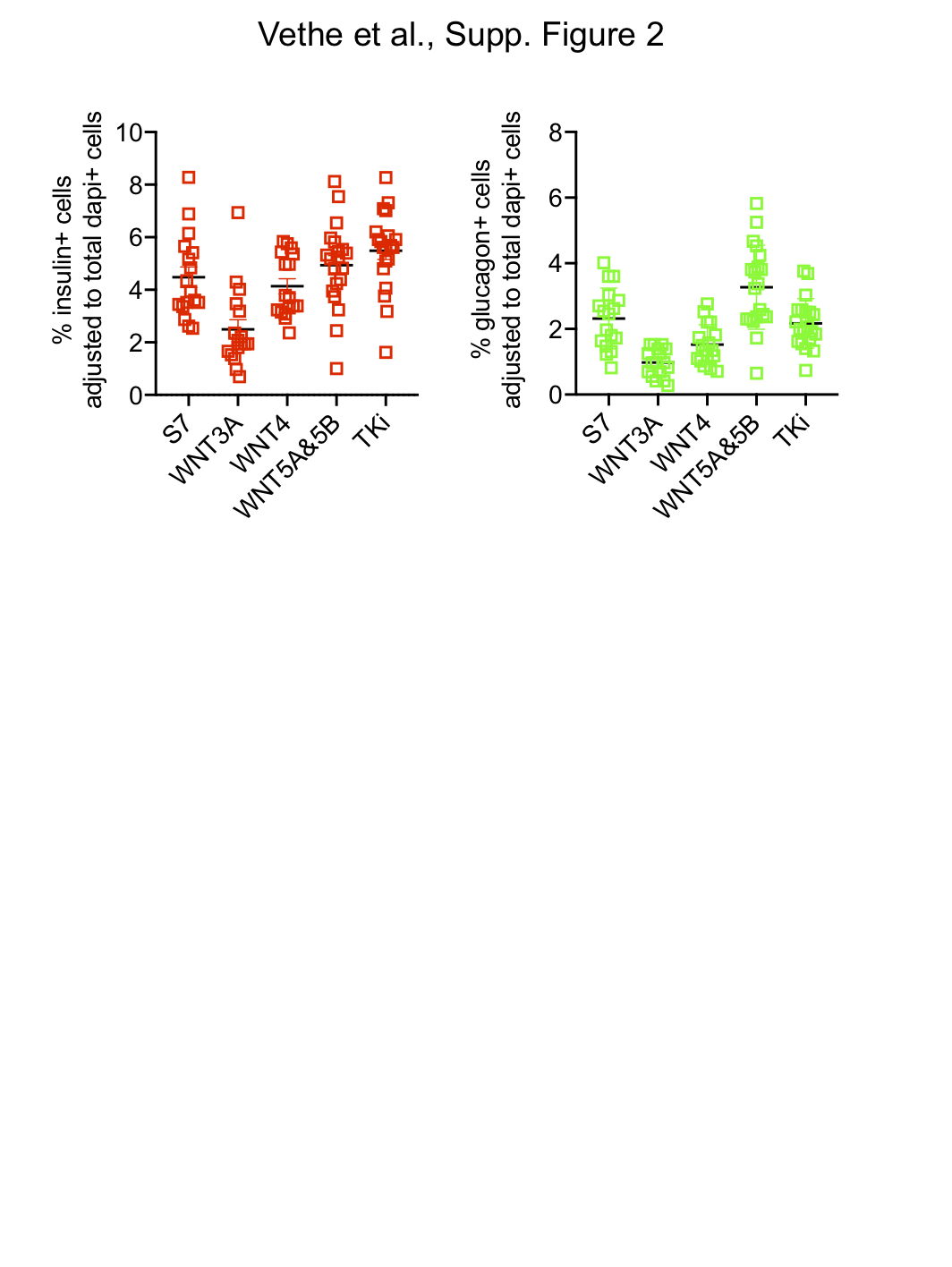

Supplement: Supplementary Figure 2 — Dapi+ cells were counted from three confocal areas for each condition to calculate the % insulin+ and % glucagon + cells as normalized to total number of dapi+ cells. [file Image_2.TIFF]
