# Supplementary material for: The Effect of Wnt Pathway Modulators on Human iPSC-Derived Pancreatic Beta Cell Maturation
Source: Front Endocrinol (Lausanne). 2019 May 8;10:293. doi: 10.3389/fendo.2019.00293 (PMC6518024; doi:10.3389/fendo.2019.00293)
Supplement: Supplementary Table 1 — The viability of S7 cells was also checked 48 h after Wnt-treatment. We chose to treat S7 cells with WNT3A (200 ng/mL), WNT4 (100 ng/mL), WNT5A (400 ng/mL), WNT5B (80 ng/mL), WNT5A&5B combination (400/ 80 ng/mL) and TKi at a concentration of 5 μmol/L). The table shows the viability of S7 cells at each of the concentrations of Wnt-modulators tested. [file Table_1.pdf]

Modulator	Concentration	Viability
WNT3A	200 ng/ mL	65%
WNT4	100 ng/ mL	77%
WNT5A	400 ng/ mL	66%
WNT5B	80 ng/ mL	71%
WNT5B	400 ng/ mL	60%
WNT5A&5B	400/ 80 ng/ mL	76%
WNT5A&5B	400/ 400 ng/ mL	80%
TKi	1 µmol/ L	53%
TKi	5 µmol/ L	73%
Untreated S7 cells	-	89%

Supplementary Table 1.

The viability of S7 cells was also checked 48 hours after Wnt-treatment. We chose to treat S7 cells with WNT3A (200 ng/mL), WNT4 (100 ng/mL), WNT5A (400 ng/mL), WNT5B (80 ng/mL), WNT5A&5B combination (400/ 80 ng/mL) and TKi at a concentration of 5 µmol/L). The table shows the viability of S7 cells at each of the concentrations of Wnt-modulators tested.
