# Supplementary material for: The Effect of Wnt Pathway Modulators on Human iPSC-Derived Pancreatic Beta Cell Maturation
Source: Front Endocrinol (Lausanne). 2019 May 8;10:293. doi: 10.3389/fendo.2019.00293 (PMC6518024; doi:10.3389/fendo.2019.00293)
Supplement: Supplementary Table 2 — Activation status of Wnt/β-catenin, Wnt/Ca2+, and Wnt/PCP pathways in TKi-treated S7 cells as compared to untreated S7 cells, The significance values for the canonical pathways is calculated using Fisher's exact test and assuming a right-tailed distribution (-log(p-value)). The significance indicates the probability of association of proteins from our dataset with proteins of the canonical pathway by chance alone. Ratio denotes here the number of proteins from our data set annotated as involved in the pathway as a fraction of all proteins annotated in the given pathway. IPA predict activated pathways (positive z-scores) or inhibited pathways (negative z-scores) based on the relative abundance of all proteins annotated in Wnt pathways from our dataset. [file Table_2.pdf]

Ingenuity Canonical Pathways	-log(p-value)	Ratio	z-score
Wnt/β-catenin signaling	4.62	0.51	-1.688
Wnt/Ca^{2+} pathway	1.56	0.48	-0.365
Wnt/PCP pathway	2.59	0.54	-1.257

Supplementary Table 2.

Activation status of Wnt/β-catenin, Wnt/Ca^{2+} and Wnt/PCP pathways in TKi-treated S7 cells as compared to untreated S7 cells, The significance values for the canonical pathways is calculated using Fisher's exact test and assuming a right-tailed distribution (-log(p-value)). The significance indicates the probability of association of proteins from our dataset with proteins of the canonical pathway by chance alone. Ratio denotes here the number of proteins from our data set annotated as involved in the pathway as a fraction of all proteins annotated in the given pathway. IPA predict activated pathways (positive z-scores) or inhibited pathways (negative z-scores) based on the relative abundance of all proteins annotated in Wnt pathways from our dataset.
